# Intestinal hemorrhage caused by Meckel’s diverticulum with ectopic gastric mucosa on polypoid lesion: a case report

**DOI:** 10.1186/s40792-016-0252-4

**Published:** 2016-11-04

**Authors:** Toshiyuki Irie, Seiichi Shinji, Hiroki Arai, Hayato Kan, Takeshi Yamada, Michihiro Koizumi, Yasuyuki Yokoyama, Goro Takahashi, Takuma Iwai, Mikihiro Okusa, Keiichiro Ohta, Eiji Uchida

**Affiliations:** Department of Gastrointestinal and Hepato-Biliary-Pancreatic Surgery, Nippon Medical School, 1-1-5, Sendagi, Bunkyo-ku, Tokyo, 113-8603 Japan

**Keywords:** Meckel’s diverticulum, Polypoid lesion, Double-balloon enteroscopy, Single-incision laparoscopy-assisted surgery

## Abstract

Meckel’s diverticulum may sometimes present as an intraluminal polypoid mass causing small bowel obstruction; however, gastrointestinal bleeding due to Meckel’s diverticulum with a polypoid lesion is rare. A 14-year-old girl presented with tarry stool and syncope in our hospital. Laboratory examination showed iron-deficiency anemia with a low hemoglobin level of 5.8 g/dl. The bleeding site was detected by neither upper gastrointestinal endoscopy nor colonoscopy. Transanal double-balloon enteroscopy showed a diverticulum with an ulceration at a site approximately 50 cm from the ileocecal valve and a polypoid lesion inside of the diverticulum. Histopathological examination of a polypoid lesion revealed an ectopic gastric mucosa of the fundic type. Furthermore, technetium-99m pertechnetate scintigraphy showed a hot spot in her lower right abdomen. On the basis of these findings, she was diagnosed as having hemorrhagic Meckel’s diverticulum. Single-incision laparoscopy-assisted segmental bowel resection of the ileum was performed. The patient recovered well, and she was discharged from the hospital on postoperative day 7. She was doing well 6 months later without evidence of reoccurrence. In this report, we describe a case of Meckel’s diverticulum with a polypoid lesion; hemorrhage may have occurred owing to the ulceration of the ileal mucosa with which the polypoid lesion directly came in contact. We consider this case to be of interest to gain insight into the site and mechanism of ulceration associated with Meckel’s diverticulum.

## Background

Meckel’s diverticulum is the most common congenital malformation of the gastrointestinal tract (present in 0.6–4 % of the population) due to the persistence of the congenital vitellointestinal duct [1]. Although it is usually asymptomatic, about 4.2–6.4 % of patients are symptomatic [2]. Complications arising from Meckel’s diverticulum include gastrointestinal bleeding, intussusception, intestinal obstruction, abdominal pain, and incarcerated hernia [3]. The main cause of bleeding is the acid secreted from the ectopic mucosa, leading to ulceration of the adjacent ileal mucosa. Furthermore, the recurrent intussusception may cause trauma, inflammation, mucosal erosion, and bleeding [1].

On the other hand, Meckel’s diverticulum may at times present as an intraluminal polypoid mass causing small bowel obstruction, and this occurs when the diverticulum is inverted into the ileum and may serve as a lead point for intussusceptions [4]. However, gastrointestinal bleeding due to Meckel’s diverticulum with a polypoid lesion is rare. In this report, we describe a case of intestinal hemorrhage caused by Meckel’s diverticulum with ectopic gastric mucosa on the polypoid lesion.

## Case presentation

**Fig. 1:**
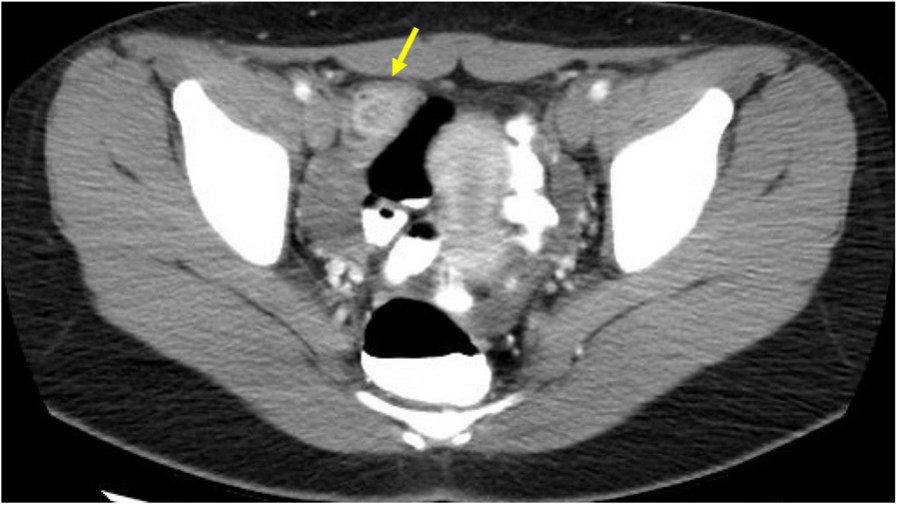
Contrast-enhanced CT images showing a tumor with a contrasting effect in the ileum (*arrow*)

**Fig. 2:**
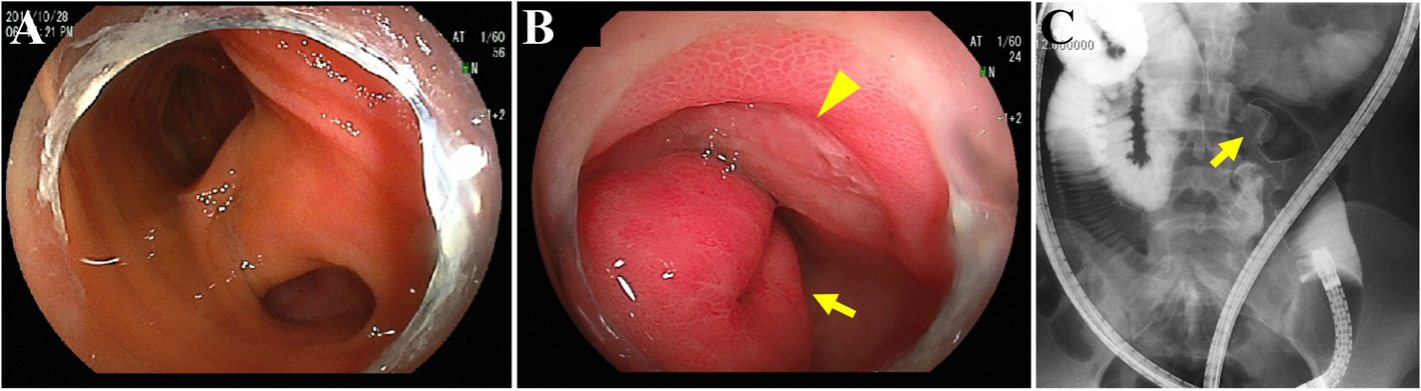
Transanal double-balloon enteroscopy revealing bifurcation of the intestinal tract (**a**). In one lumen seen at the bottom of the screen, there was a polypoid lesion inside of the diverticulum (**b**, *arrow*) and ulceration (**b**, *arrowhead*). A small bowel series also showed a polypoid lesion inside of the diverticulum at a site approximately 50 cm from the ileocecal valve (**c**, *arrow*)

**Fig. 3:**
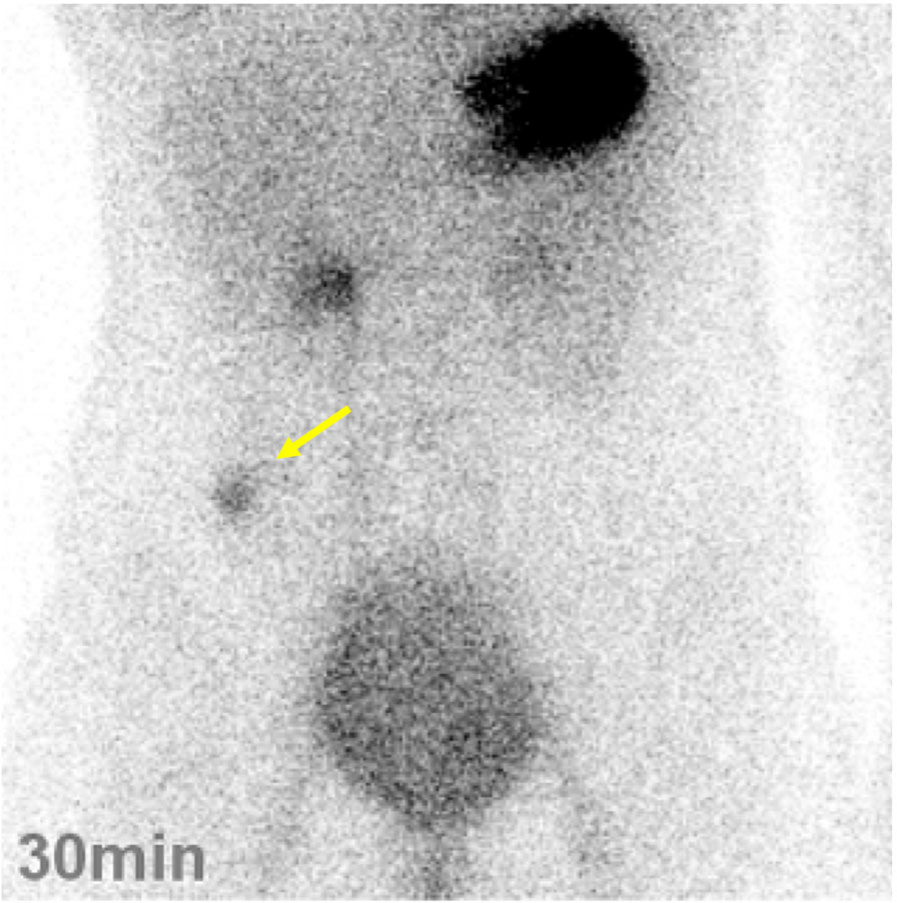
Technetium-99m pertechnetate scintigraphy image showing a hot spot in her lower right abdomen (*arrow*)

A 14-year-old Japanese girl experienced passing tarry stool and syncope twice since October 2015. She visited a pediatric clinic. A laboratory examination showed iron-deficiency anemia with a low hemoglobin level of 5.8 g/dl. She visited our hospital for a more detailed examination. Physical examinations showed an anemic change in the palpebral conjunctiva. On admission, her blood pressure was 98/58 mmHg, heart rate was 98 beats per minute, and respiratory rate was 18 breaths per minute. Her consciousness level was normal. Laboratory tests (Table 1) showed the following blood and biochemical findings: hemoglobin, 6.2 g/dl (normal, 12–16 g/dl); serum iron, 10 μg/dl (normal, 70–180 μg/dl); unsaturated iron-binding capacity, 386 μg/dl (normal, 137–325 μg/dl); and ferritin, 5.8 μg/dl (normal, 6.2–138.0 μg/dl). Her contrast-enhanced CT images showed a tumor with a contrasting effect in the ileum (Fig. 1, arrow). Neither upper gastrointestinal endoscopy nor colonoscopy detected the source of bleeding from ulceration and neoplastic lesion. Transanal double-balloon enteroscopy revealed bifurcation of the intestinal tract (Fig. 2a), and in one lumen seen at the bottom of the screen, a polypoid lesion inside of the diverticulum (Fig. 2b, arrow) and ulceration (Fig. 2b, arrowhead) were observed. A small bowel series also showed a polypoid lesion inside of the diverticulum with at a site approximately 50 cm from the ileocecal valve (Fig. 2c, arrow). Histopathological examination of a polypoid lesion specimen revealed an ectopic gastric mucosa. We tattooed the intestinal mucosa adjacent to the diverticulum for marking. Furthermore, technetium-99m pertechnetate scintigraphy showed a hot spot in her lower right abdomen (Fig. 3, arrow). On the basis of these findings, she was diagnosed as having hemorrhagic Meckel’s diverticulum.

**Table 1:** Laboratory findings on admission

WBC	7500/μl
RBC	210 × 10^4^/μl
Hb	6.2 g/dl
Ht	18.7%
Plt	41.8 × 10^4^/μl
PT	13.4 s
PT-INR	1.13
APTT	27.8 s
Fe	10 μg/dl
UIBC	386 μg/dl
Ferritin	5.8 μg/dl
AST	20 IU/l
ALT	20 IU/l
LDH	156 IU/l
ALP	209 IU/l
CK	81 IU/l
T-Bil	0.3 mg/dl
Na	138 mEq/l
Cl	103 mEq/l
K	4.1 mEq/l
BUN	8.6 mg/dl
Cr	0.55 mg/dl
TP	6.9 g/dl
Alb	4.3 g/dl
CRP	0.02 mg/dl

**Fig. 4:**
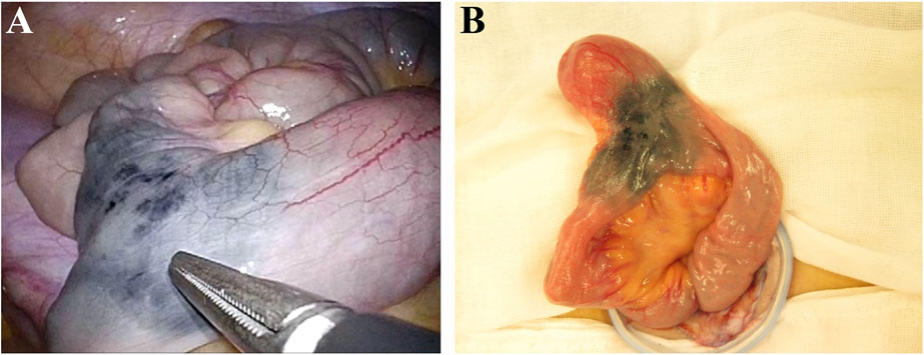
Diverticulum located on the antimesenteric border of the ileum (**a**) and extraction of the bowels outside of the body (**b**)

Single-incision laparoscopy-assisted surgery was performed. By examining the abdominal cavity, we easily found the diverticulum located on the antimesenteric border of the ileum (Fig. 4a) and extracted the bowels outside of the body (Fig. 4b). Under direct open visualization from the umbilical wound, segmental bowel resection of the ileum and functional end-to-end anastomosis were carried out. The operation time was 100 min, and blood loss was negligible.

**Fig. 5:**
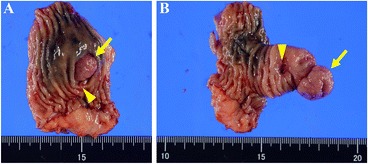
Surgically resected specimen demonstrating a pedunculated polyp in the diverticulum (**a, b**, *arrow*). When the diverticulum was retracted, we observed the ulceration adjacent to the polypoid lesion (**a, b**, *arrowhead*)

**Fig. 6:**
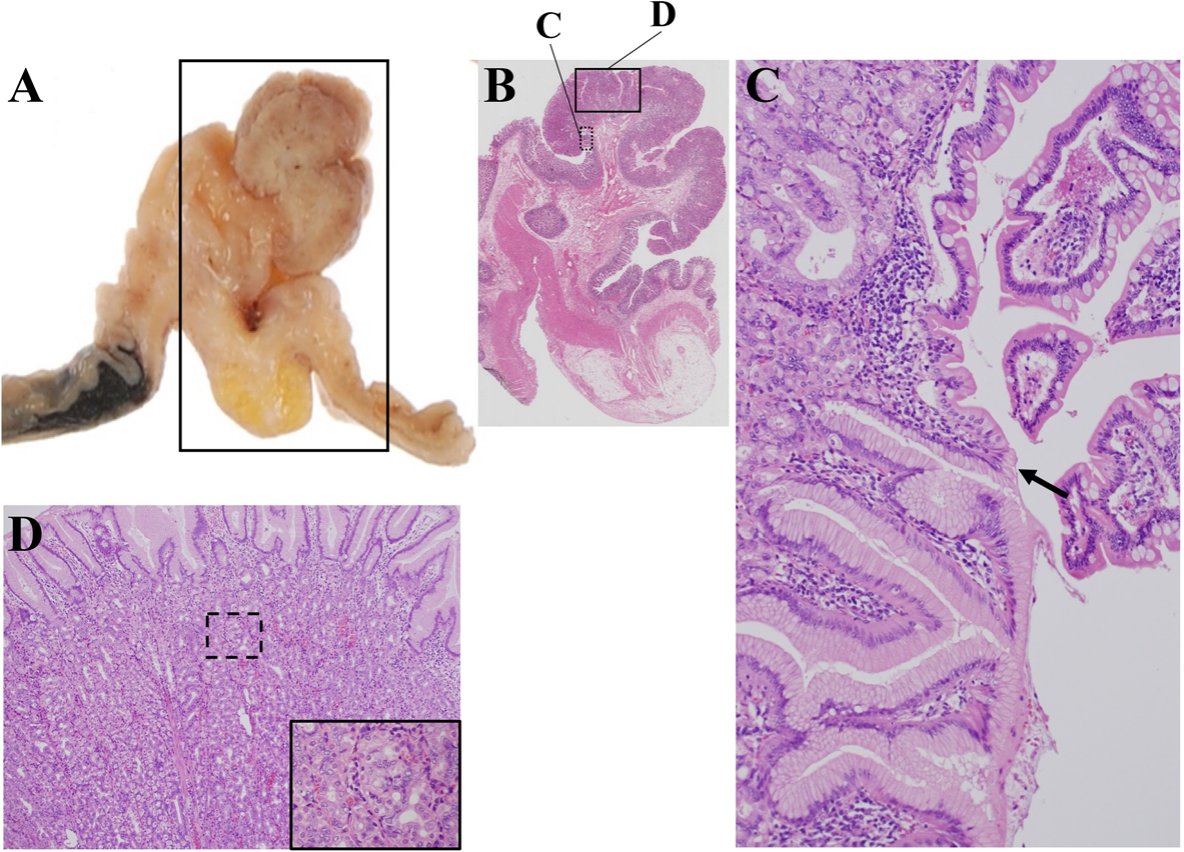
Histological appearance of the polypoid lesion specimen (**a**, **b**) showing a transition between the ectopic gastric mucosa and the intestinal mucosa (**c**, *arrow*). Ectopic gastric mucosa of the fundic type (**d**)

**Fig. 7:**
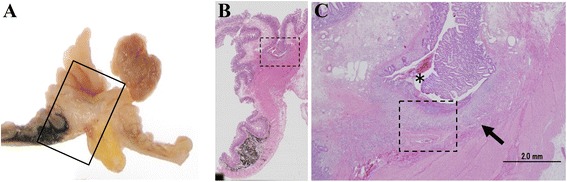
Superficial ulceration lesion specimen (**a**, **b**) showing lack of mucosal epithelium and infiltration of inflammatory cells immediately under the polypoid lesion (**c**, *arrow*). Bleeding was observed on the surface of the erosion (**c**, *asterisk*), which was considered to be the source of bleeding

The surgically resected specimen demonstrated a pedunculated polyp in the diverticulum (Fig. 5a, b, arrow). When the diverticulum was retracted, we observed the ulceration adjacent to the polypoid lesion (Fig. 5a, b, arrowhead). The histological appearance of the polypoid lesion (Fig. 6a, b) showed a transition between the ectopic gastric mucosa and the intestinal mucosa (Fig. 6c, arrow), and the ectopic gastric mucosa was of the fundic type (Fig. 6d). On the other hand, a specimen of the superficial ulceration (Fig. 7a, b) showed a lack of the mucosal epithelium and infiltration of inflammatory cells immediately under the polypoid lesion (Fig. 7c, arrow). And bleeding was also observed on the surface of the erosion (Fig. 7c, asterisk), which was considered to be the source of bleeding. The patient did well postoperatively, and she was discharged from the hospital on day 7 without the need for analgesics or any complications. She was doing well 6 months later without evidence of reoccurrence.

## Discussion

Meckel’s diverticulum is the most common congenital malformation of the small intestine. Clinical symptoms can mostly be related to a heterotopic tissue in the diverticulum and abnormal fixation of the tip of the diverticulum. Meckel’s diverticulum is diagnosed using multiple modalities such as conventional radiography, barium studies, ultrasonography, computed tomography, scintigraphy, digital subtraction angiography, laparotomy, capsule endoscopy, and double-balloon enteroscopy (DBE) [4, 5]. However, it is known to both radiologists and clinicians that the preoperative diagnosis of symptomatic Meckel’s diverticulum is difficult [1]. Our case was diagnosed by DBE and technetium-99m pertechnetate scintigraphy. DBE is an excellent diagnostic modality because direct observation of both Meckel’s diverticulum and ulceration and histopathological diagnosis by endoscopic biopsy are possible [5]. However, DBE should be used complementarily with other less invasive examinations such as technetium-99m pertechnetate scintigraphy to confirm or establish the diagnosis [5]. We also performed technetium-99m pertechnetate scintigraphy as a supplemental examination, which showed a hot spot in her lower right abdomen. It is a noninvasive examination specifically of ectopic gastric mucosa and not Meckel’s diverticulum, and is helpful in determining the approximate location of a lesion. Furthermore, it is a useful diagnostic method for pediatric patients because it has a higher sensitivity in the range of 85–90 % in children than in adults (approximately 60 %) [6].

Sagar et al. reported that bleeding from Meckel’s diverticulum due to ectopic gastric mucosa is the most common clinical presentation particularly in young patients [1]. Ectopic gastric and pancreatic tissues frequently found in the diverticulum are the primary causes of gastrointestinal bleeding, because highly acidic secretions from gastric tissues and alkaline secretions from pancreatic tissues cause ulcerations of the adjacent normal ileal mucosa [7]. With regard to the incidence of small bowel tumors, according to a report of analysis of a prospectively collected database of double-balloon enteroscopy, of the 1106 patients who underwent double-balloon enteroscopy procedures, 134 (12.1 %) were reported to have a small bowel tumor, of whom 36 (26.9 %) had obscure gastrointestinal bleeding and 20 (14.9 %) had overt gastrointestinal bleeding [8]. Sometimes, Meckel’s diverticulum may present as an intraluminal polypoid mass causing small bowel obstruction when the diverticulum is inverted into the ileum. In our case, we found a polypoid lesion inside of Meckel’s diverticulum with ectopic gastric mucosa which caused gastrointestinal bleeding. By searching PubMed (www.ncbi.nlm.nih.gov/PubMed, from 1977 to Dec 2015) using a combination of MeSH terms including “Meckel’s diverticulum” and “polyp”, we found only two reported cases of Meckel’s diverticulum with polypoid lesions causing gastrointestinal bleeding diagnosed by capsule endoscopy [9, 10]. In our case, transanal DBE revealed a polypoid lesion inside of the diverticulum and an intestinal ulceration in direct contact with the polypoid lesion. Furthermore, the surgically resected specimen showed a pedunculated polyp in the diverticulum with the ulceration adjacent to the polypoid lesion. Histopathologically, ectopic gastric mucosa existed at the head of the polypoid lesion, indicating that the ulceration was caused by exposure of the ectopic gastric mucosa not by mechanical stimulation or ischemia due to intussusception. How a polypoid lesion in Meckel’s diverticulum develops is unknown. It has been suggested that chronic abnormal peristaltic movements caused by the ectopic tissue at the bottom of Meckel’s diverticulum may secondarily lead to the formation of a polypoid lesion. Moreover, the elevated ectopic gastric mucosa may be easily exposed to the adjacent small intestinal mucosa, formation of ulcer.

Surgical management, including diverticulectomy or segmental bowel resection and anastomosis, is required for Meckel’s diverticulum, and laparoscopic excision has been increasingly used owing to the advances in the development of minimally invasive surgery in children [11]. Furthermore, single-incision laparoscopic techniques have recently been adopted as a laparoscopy-assisted surgical option, which would be otherwise required for specimen extraction, and are potentially associated with the decrease in port-site- and incision-related morbidity, reduced postoperative pain, and improved cosmetic features [12]. In our present case, single-incision laparoscopy-assisted umbilical minilaparatomy was performed, which enabled the examination of the abdominal cavity, easy detection of the lesion, bowel extraction outside of the body, and segmental bowel resection via the smallest possible wound required for anastomosis.

## Conclusions

We experienced treating a rare case of Meckel’s diverticulum with a polypoid lesion, and hemorrhage may have occurred owing to the ulceration of the ileal mucosa with which the polypoid lesion directly came in contact. We consider this case to be of interest to gain insight into the site and mechanism of ulceration associated with Meckel’s diverticulum.

## Abbreviations

DBE: Double-balloon enteroscopy
